# IFEM white paper on gender diversity and inclusion

**DOI:** 10.1007/s43678-022-00379-w

**Published:** 2022-08-30

**Authors:** Gayle Galletta, Cherri Hobgood, Gillian Sheppard, Ffion Davies, Priyadarshini Marathe, Sally McCarthy, Imron Subhan, Heike Geduld

**Affiliations:** 1 Department of Emergency Medicine, University of Massachusetts, Worcester, MA USA; 2 Department of Emergency Medicine, University of North Carolina, Chapel Hill, NC USA; 3 Discipline of Emergency Medicine, Memorial University of Newfoundland, St. John’s, NL Canada; 4 Department of Emergency Medicine, University Hospitals of Leicester, NHS, Leicester, England, UK; 5 Department of Emergency Medicine, Oxford University Hospitals, NHS, Oxford, England, UK; 6 Department of Emergency Medicine, Prince of Wales Hospital, Randwick, NSW Australia; 7 Department of Emergency Medicine, Apollo Hospitals, Hyderabad, Telangana India; 8 Division of Emergency Medicine, Stellenbosch University, Stellenbosch, Western Cape South Africa

## Background

The International Federation for Emergency Medicine (IFEM) is a federation of over 70 national and regional emergency medicine organizations whose mission is to advance the growth of high-quality emergency medical care through education and standards, to lead the collaboration and networking necessary to establish universal equality in service and care, and to promote the creation and growth of the specialty of emergency medicine (EM) in every country.

IFEM is also committed to promoting gender equity worldwide. The purpose of this document is to outline what IFEM will do to advance gender equity and remove barriers that may prevent women’s participation and advancement in the practice of EM and in IFEM leadership roles.

IFEM acknowledges that gender disparities in medicine are widespread and vary among countries. There are three ways of looking at this:

- Proportion of women in the medical workforce
- Proportion of women in senior leadership positions within their field
- Perceptions and attitudes of co-workers and patients toward female doctors

### Proportion of women in the medical workforce

From a global perspective, it is important to note that the percentage of female physicians in general varies widely, from 20% in Japan and Korea to 77% in Latvia and Estonia. For many areas of the world, there are either no data or no reliable data regarding the number of women working specifically in EM, although it is estimated to be approximately 33%.

### Women in senior leadership positions

Women are notoriously underrepresented in EM leadership positions. It is acknowledged that there is a “leaky pipe” phenomenon, where women drop off as one moves up the leadership ladder. Senior leadership positions include senior clinical roles (department chair, operations officers, board members) and senior academic roles (associate and full professor). Reasons cited for this attrition of women at each step of the career ladder include the “lack of fit” model, where inconsistency between stereotypes about women and perceived requirements for success in male-typed positions leads to the perception that women are not well suited for them, producing negative expectations about their likely performance. These expectations in turn lead to the presumption that women lack the competence necessary to do well in these positions and are unlikely to succeed. This contributes to and compounds less mentoring and fewer role models, lack of managerial support, lack of opportunities and recognition, negative performance assessments, and challenges with work-life integration. Lack of role models may prevent women not only from pursuing leadership positions, but also from entering the specialty entirely. Sexism and a “male-dominated work environment” (numerical male dominance, where policies and informal practices are dictated by men and detrimental to women’s personal and organisational wellbeing) perpetuate a culture of systemic bias. This embedded and difficult to identify system of bias, produces a workplace that is advantageous toward men and filled with hidden barriers for women.

In addition, it is virtually universal, due to entrenched and persistent gender stereotyping within societies, that women are responsible for the majority of household chores and childcare. Gender stereotypes can be challenged by providing “parental” or “caregiver” leave and flexible work arrangements to men and women alike, questioning the assumption that a distinct division of gender-based roles exists. This has long been the case in Scandinavia. In many countries, however, gender stereotyping is one of the reasons why women are more likely to work part-time and less likely to take on leadership positions. Women in EM, especially those in early to mid-career, have also been more heavily burdened with parental responsibilities brought upon by the pandemic. There is broad agreement that female EM physician mothers need more support to achieve academic success due to a “motherhood penalty”, which is also driven by assumptions that a woman will be less reliable, less committed, less competent, and less interested in her career than she was prior to becoming a mother. These gender inequities in EM contribute to the well-recognized gender pay gap and burnout among physicians.

### Perception and attitudes of co-workers and patients toward female doctors

Female physicians provide excellent care, sometimes better than their male counterparts. Despite this, female emergency medicine physicians are often mistaken as nurses by their patients and not addressed as “doctor” by their colleagues when giving presentations. In Sri Lanka, the word “doctor” refers only to men, therefore, female physicians are addressed as “Dr. (Mrs.)”. Nurses may also be less likely to cooperate with female physicians and studies have shown that female EM trainees receive lower evaluations despite scoring equally high on exams. This is likely due to “attribution bias” where, for the same behavior, men are perceived as confident and women as bossy. Women must, therefore, work harder and smarter than their male colleagues since their competency and contribution are often undervalued or overlooked. Achieving gender equity also benefits men’s health and wellbeing, and creates more equitable societies, workplaces, and relationships for all genders. A recurrent request from a diverse sampling of countries is the need for male allies within EM. Men in leadership positions must be willing to share these positions with their female colleagues. In addition, all leaders should be encouraged to take a woman along with them as they climb the leadership ladder and support one another to function. To advance in leadership positions, EM physicians, especially those in training and early career, need sponsorship, not merely mentorship. Sponsorship requires more active and intentional initiatives than mentorship and includes advocating, networking, and making connections.

## Current status

The Gender Specific Issues Special Interest Group (GSI-SIG) of IFEM was founded in 2014 to provide an expert forum to discuss a variety of matters related to gender-specific issues within the specialty of emergency medicine and emergency patient care, acknowledging that each country is on its own trajectory toward gender equity, and not every solution will work in every country. Gender Equity and Equality (GEE) Workshops have been developed to deliver training and awareness according to local needs and IFEM has endorsed gender equity and equality activities within conferences and meetings. To ensure that women are being given equitable speaking opportunities at their conferences, IFEM performed a self-assessment of the gender distribution of speakers from its three most recent pre-pandemic International Conferences of Emergency Medicine held in Cape Town, South Africa in 2016; Mexico City, Mexico in 2018; and Seoul, South Korea in 2019. Women comprised 25% of organizing committees, 31% of plenary speakers, and 22% of invited speakers. With intention, ICEM 2022, held in Melbourne, Australia, achieved gender parity among invited speakers for the first time.

## IFEM pledge

While IFEM cannot control gender inequities in emergency medicine in individual countries, we can pledge to do our part and set an example. Specific actions are:

- Recognize that implicit gender bias and sexism exist for both physicians and patients in emergency medicine
- Collect and publish data relating to IFEM leadership positions and awards
- Develop a leadership mentorship program to encourage more women to apply for leadership positions
- Ensure gender balanced and informed ICEM organizing committees and speaker ratios of at least 40% for each gender
- Continuing to develop and support Gender Equity and Equality (GEE) workshops throughout the world to raise awareness and provide training tools to understand the basis of gender differences and discrimination and provide resources to enable gender equity in the emergency department
- Identify and highlight risk and protective factors for emergency medicine work environments seeking to promote gender equity
- Encourage the establishment of gender-specific issues committees within each IFEM member society to ensure global cooperation and representation within IFEM’s GSI-SIG
- Support member organizations in their efforts to ensure that women are represented within their national organizations

By removing barriers that prevent women from practicing emergency medicine and leading our specialty, IFEM hopes to increase the number of women emergency medicine physicians to better reflect our patients’ diversity and improve emergency care for the patients that we serve globally.

The unabridged white paper and references can be accessed on the International Federation for Emergency Medicine website: https://www.ifem.cc/white_paper_on_gender_diversity_and_inclusion.

